# Volume-Associated Clinical and Histopathological Effects of Intranasal Instillation in Syrian Hamsters: Considerations for Infection and Therapeutic Studies

**DOI:** 10.3390/pathogens11080898

**Published:** 2022-08-10

**Authors:** Catalina Forero, Jana M. Ritter, Josilene Nascimento Seixas, JoAnn D. Coleman-McCray, Marie Brake, Jillian A. Condrey, Cassandra Tansey, Stephen R. Welch, Sarah C. Genzer, Jessica R. Spengler

**Affiliations:** 1 Comparative Medicine Branch, Division of Scientific Resources, Centers for Disease Control and Prevention, Atlanta, GA 30329, USA; 2 Infectious Diseases Pathology Branch, Division of High-Consequence Pathogens and Pathology, Centers for Disease Control and Prevention, Atlanta, GA 30329, USA; 3 Viral Special Pathogens Branch, Division of High-Consequence Pathogens and Pathology, Centers for Disease Control and Prevention, Atlanta, GA 30329, USA

**Keywords:** Syrian hamster, animal model, respiratory virus model, intranasal instillation, intranasal inoculation, histopathology

## Abstract

Syrian hamsters are a key animal model of SARS-CoV-2 and other respiratory viruses and are useful for the evaluation of associated medical countermeasures. Delivery of an infectious agent or intervention to the respiratory tract mirrors natural routes of exposure and allows for the evaluation of clinically relevant therapeutic administration. The data to support instillation or inoculation volumes are important both for optimal experimental design and to minimize or avoid effects of diluent alone, which may compromise both data interpretation and animal welfare. Here we investigate four intranasal (IN) instillation volumes in hamsters (50, 100, 200, or 400 µL). The animals were monitored daily, and a subset were serially euthanized at one of four pre-determined time-points (1, 3, 7, and 14 days post-instillation). Weight, temperature, oxygen saturation, CBC, radiographs, and respiratory tissue histopathology were assessed to determine changes associated with instillation volume alone. With all the delivery volumes, we found no notable differences between instilled and non-instilled controls in all of the parameters assessed, except for histopathology. In the animals instilled with 200 or 400 µL, inflammation associated with foreign material was detected in the lower respiratory tract indicating that higher volumes may result in aspiration of nasal and/or oropharyngeal material in a subset of animals, resulting in IN instillation-associated histopathology.

## 1. Introduction

Intranasal (IN) instillation in laboratory animal species is a commonly used approach in biomedical research in the development of animal models of disease and in the evaluation of therapeutic and vaccine candidates. It has been successfully used to evaluate the efficacy of targeted challenges or therapies for either the upper respiratory tract or lungs in research mice for several decades [1,2,3,4]. Instillation is less resource demanding and technically challenging when compared to the alternatives, including intubation or aerosol generators.

The distribution of IN-instilled innocula and the effects of volume, time, body position, and anesthesia on this delivery method have been studied in mice [5,6] and other species, such as ferrets [7], cotton rats [8], and NHPs [8]. Although mice are frequently used to study pulmonary infectious diseases and for other non-infectious research applications, other rodents have proven to be more effectual for use in certain disease models, such as guinea pigs for tuberculosis [9] and Syrian hamsters for other pathogens, including SARS-CoV-2 [10,11,12]. The use of hamsters has more than doubled in the last two decades [13], most notably to model respiratory pathogens and test interventions for specific agents. In addition to work on SARS-CoV-2, hamsters are key disease models for other highly pathogenic viruses, including henipaviruses (Nipah and Hendra; [14,15,16]), and human influenza virus [17], supporting the critical role of hamsters in biomedical research.

Despite the recent surge in hamster use, most prominently in COVID-19 studies, there is a dearth of research investigating the kinetics and putative physiological and pathological effects of the IN-instillation approach to infection or the delivery of therapeutics in hamsters. The experimental studies in hamsters using IN inoculation are often guided by reports in mice [18,19]. Establishing volumetric parameters for use in hamsters would provide data to support safe and allowable intranasal delivery volumes in the species, identify the pathological effects that are independent of agent or therapeutic protocols, and highlight the clinical considerations when upper volume limits are used.

Here, we explore the volumetric-associated effects of IN instillation in Syrian hamsters on clinical presentation, oxygen saturation, CBC, radiology, and histopathology, using Dulbecco’s modified Eagle’s medium (DMEM), a common diluent for virus stocks. The volumes we investigated (50–400 µL) matched or exceeded those previously used in hamsters; with reported instillation volumes ranging from 20 µL [20] to 100 µL [17,21,22,23,24]. The volumes were selected based on the literature for mice and hamster pulmonary parameters, including lung capacity, mechanics, and function [25,26,27,28,29,30], with the aim of determining a maximum delivery volume that can be safely administered to prevent non-specific clinical or pathological changes, and minimize pain and distress. Based on the literature and volumes selected, we hypothesized that the clinical analyses and imaging of the hamsters would be largely unremarkable throughout the study; however, volume-dependent histological changes may be evident in the experimental groups instilled with larger volumes.

## 2. Results

### 2.1. No Volume-Associated Changes Were Seen in Clinical Measures, Oxygenation, or Pulmonary Radiography in Intranasally Instilled Hamsters

**Figure 1:**
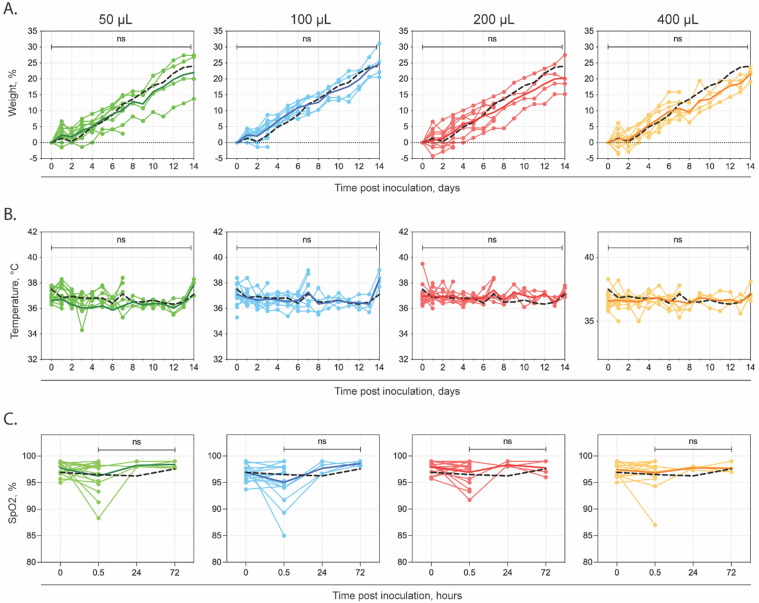
Changes in weight, temperature and SpO_2_ in Syrian hamsters following intranasal instillation with DMEM alone. Groups of 5- to 6-week old Syrian hamsters were intranasally instilled with 1 of 5 volumes of sterile DMEM (0, 50, 100, 200 or 400 µL) and serially euthanized at 1, 3, 7, or 14 days post-instillation. (**A**) Mean change in weight (compared to baseline at day 0) and (**B**) mean temperatures; (**C**) Pulse oximetry readings taken under anesthesia pre- and post-instillation and prior to euthanasia for subset euthanized at 1 and 3 days post-instillation. Individual values are shown, with solid lines representing mean value at each time point (50 µL—green; 100 µL—blue; 200 µL—red; 400 µL—orange). In addition, the mean values for the non-instilled control animals are represented (0 µL—black dashed line). ns—not significant compared to non-instilled controls.

No differences in weight, body temperature, or oxygen saturation were observed between the experimental groups. All of the animals showed a consistent weight gain throughout the study, and no statistical differences were seen in the growth rate of the animals between the instillation volume groups, or as compared to the controls (Figure 1A). Other than a mild drop in weight (2–3 g) seen in all of the animals (experimental groups and controls) at 2 days post-instillation (dpi), throughout the course of the study all of the animals showed an upward trend consistent with the expected growth for this age cohort. The body temperature was monitored daily (between 0900–1100) with microchip transponders (Figure 1B). The temperature ranges remained consistent between ~36–38 °C, and no statistical differences were seen between the groups. The animals designated for each timepoint were separated from their home cage and transported to the study room, prior to final weight and body temperature readings. During transport, the animals became highly active within the transport cage. The temperatures for the transported animals were 1–2 °C higher than those that remained in their home cage. The animals measured from their home cage were usually asleep prior to temperature readings. At earlier timepoints, the effect of transport on body temperature was not evident, as a large proportion of the groups were assessed in home cages. However, at 7 dpi and 14 dpi, when half or all the animals were assessed after transport, there was a noted mean increase in body temperature for all of the groups. These changes were consistent across the groups and are believed to solely reflect handling conditions. The oxygen saturation prior to intranasal instillation showed no statistical differences between all of the groups (Figure 1C). At the 30-min time point (after instillation) at 0 dpi, some of the animals displayed a significant drop in their oxygen saturation, equally affecting all of the groups. This was attributed to the animal’s individual movement between stations (weight, microchipping, instillation stations) as they were moved from one nose cone to the next, therefore their access to oxygen and isoflurane was intermittently disrupted. The affected animals quickly normalized after 1 min of consistent flow-by oxygen and isoflurane; these data were not recorded to maintain consistency among all of the groups and data acquisition.

**Figure 2:**
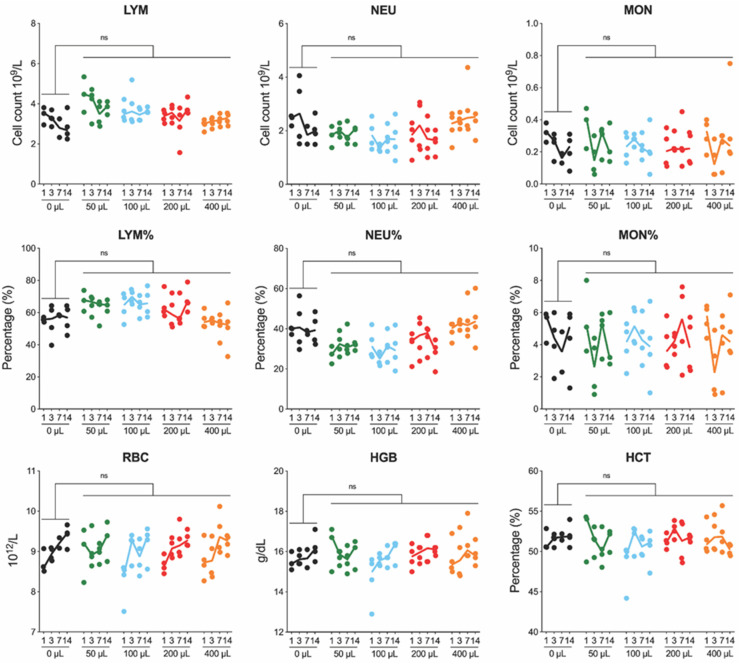
Changes in complete blood counts in Syrian hamsters after intranasal instillation with DMEM alone. Complete blood counts (CBC) performed on whole blood collected in EDTA via terminal intracardiac sampling on animals serially euthanized at 1, 3, 7, or 14 days post-instillation. Individual values shown with solid lines representing median value at each time point (0 µL—black; 50 µL—green; 100 µL—blue; 200 µL—red; 400 µL—orange). LYM, lymphocyte; NEU, neutrophil; MON, monocyte; RBC, red blood cell; HGB, hemoglobin; HCT, hematocrit; ns, not significant.

**Figure 3:**
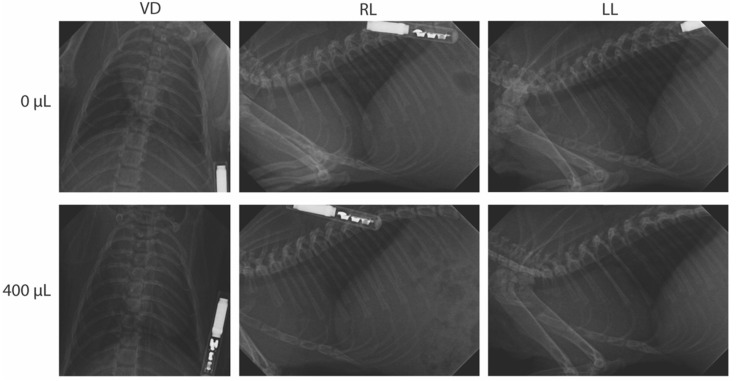
Radiographic investigation of Syrian hamsters following intranasal instillation. Groups of 5- to 6-week old Syrian hamsters were intranasally instilled with 1 of 5 volumes of sterile DMEM (0, 50, 100, 200 or 400 µL) and serially euthanized at 1, 3, 7, or 14 days post-instillation. Three-view radiographic images (ventrodorsal (VD), right lateral (RL) and left lateral (LL)) were taken per animal prior to euthanasia. Shown are representative images from non-instilled controls (0 µL) and an animal instilled with 400 µL, both at 1 day post-instillation. No notable radiographic differences were seen in instilled animals as compared to non-instilled animals.

The instillation volume-associated differences in the complete blood count or radiography were also not observed. A review of the complete blood-count values revealed no clinically relevant abnormalities in all of the parameters measured within our study (Figure 2). The neutrophils were elevated for two animals in the 0 µL group at 3 dpi, but these were not statistically significant. Finally, a review of the radiographic images by two veterinarians revealed no overt abnormalities (Figure 3). Variation between the animals was within the normal limits and was not associated with the volume of intranasal instillation. 

### 2.2. Volume-Dependent Histopathological Evidence of Foreign Material Aspiration in Intranasally Instilled Hamsters

**Figure 4:**
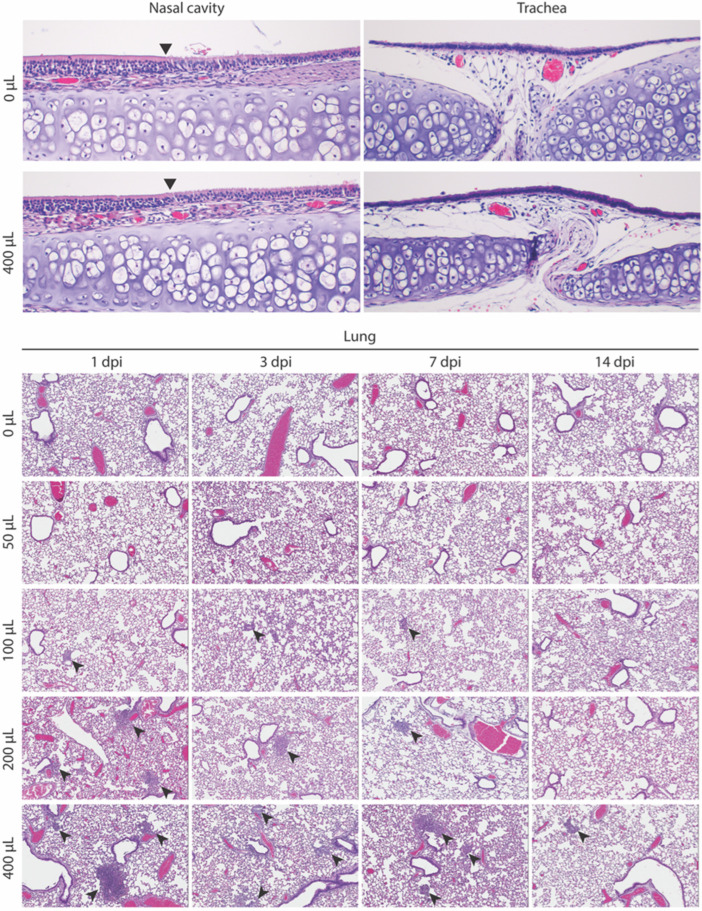
Histopathology of upper and lower respiratory tract in Syrian hamsters following intranasal instillation. Representative images from groups of 5- to 6-week old Syrian hamsters intranasally instilled with 1 of 5 volumes of sterile DMEM (0, 50, 100, 200, or 400 µL) and serially euthanized at 1, 3, 7, or 14 days post-instillation (dpi). Nasal mucosa (0 µL and 400 µL at 1 dpi; top left), arrowhead indicates transition from olfactory to respiratory epithelium. No inflammation and no epithelial changes were observed in any animals. Trachea (0 µL and 400 µL at 1 dpi; top right); intact epithelium and minimal lymphocytes were observed in subepithelial stroma. Hamster lungs after intranasal diluent instillation (lower panel). Arrows indicate inflammatory aggregates, which are variably associated with foreign material. All images: hematoxylin–eosin. Original magnifications: nasal and trachea ×200; lung ×80.

No significant gross findings were observed in the respiratory tracts of the animals at necropsy. The nasal cavity, trachea, and all lung lobes from each animal were examined microscopically. The nasal cavities had variable submucosal congestion across all of the dose groups and timepoints; no significant inflammation or epithelial degeneration was seen in any animal (Figure 4). The tracheas had no to minimal lymphoplasmacytic subepithelial infiltrates, variably present among all of the groups and not associated with dose or timepoint. 

**Figure 5:**
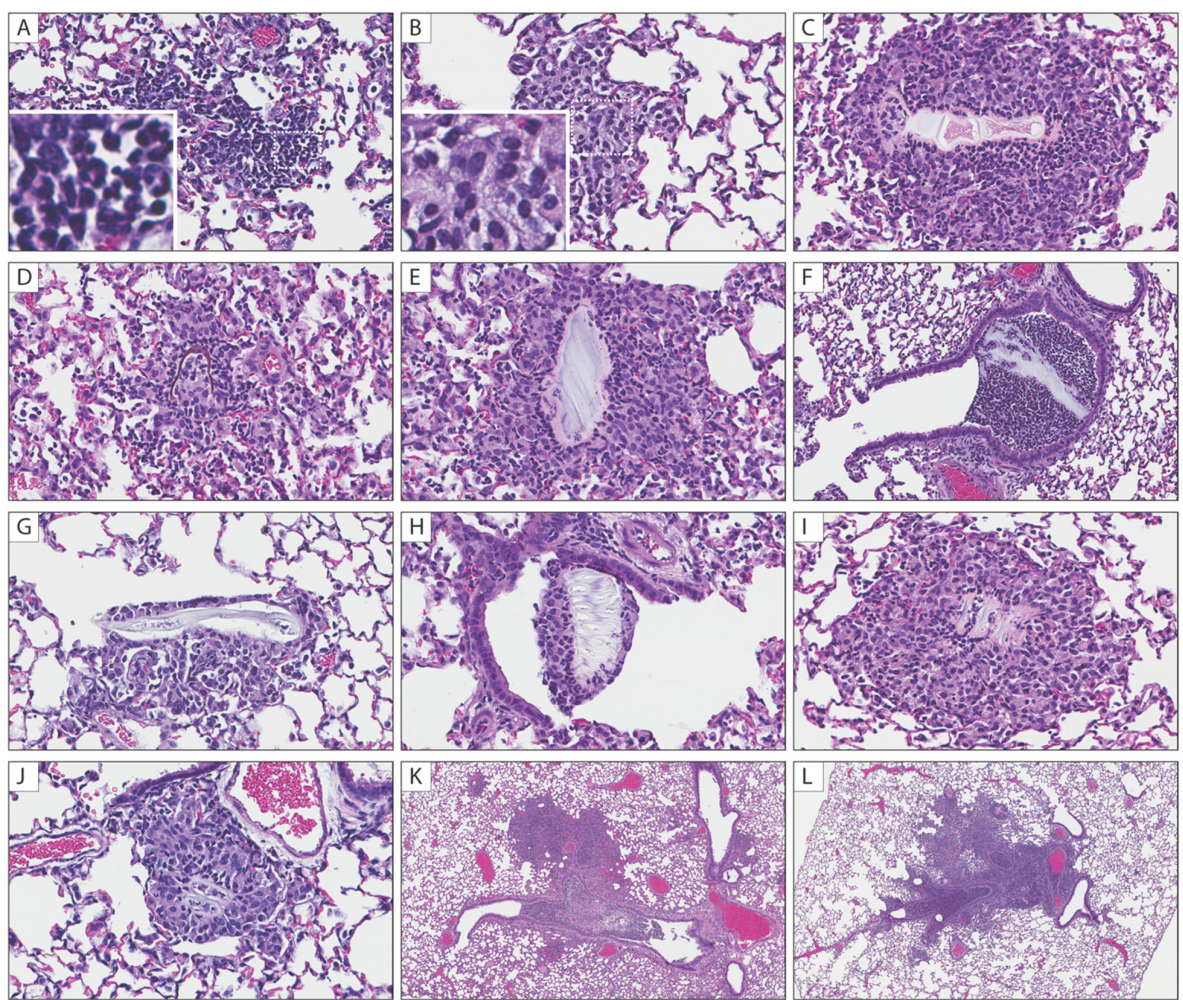
Histopathological findings in lungs of Syrian hamsters following intranasal instillation. Intranasal instillation volume-associated inflammatory foci in the terminal bronchioles and alveoli were consistently observed in 200 and 400 µL groups. (**A**) Inflammatory aggregates comprise mostly neutrophils at 1 dpi (inset: neutrophils with multilobulated nuclei); (**B**) Inflammatory aggregates comprise mostly macrophages at 3 dpi (inset: macrophages with abundant foamy cytoplasm); (**C**–**J**) Various types of aspirated foreign material associated with neutrophilic and macrophagic infiltrates in alveolar spaces and terminal bronchioles; (**K**,**L**) Focal bronchopneumonia in a 200 µL at 1 dpi (**K**) and 400 µL at 7 dpi (**L**). All images: hematoxylin–eosin. Original magnifications: (**A**–**J**) ×400; (**K**,**L**) at ×40.

The lungs from the uninoculated animals and from the 50 µL dose group had no inflammatory or other changes at any timepoint. The lungs from the animals instilled with 100 µL, 200 µL, or 400 µL diluent had a volume-associated increasing incidence of inflammatory foci in the terminal bronchioles and alveoli, often centered on variable types and amounts of foreign material (Table 1). For the 100 µL dose group, one of four (25%) animals at each timepoint had one–three very small, rare foci; only one had visible foreign material. For the 200 µL dose group, the lungs from two–four animals per timepoint, and 12 of 16 (75%) animals in total had similar foci, with foreign material identified in 7 of 12 (58%) of the affected lungs. For the 400 µL dose group, three–four animals per timepoint, and 15 of 16 (94%) animals in total had similar but more frequent foci, with foreign material identified in 8 of 15 (53%) of the affected lungs. For the 200 µL dose group, small inflammatory foci were decreased to absent or minimal (one–two small foci) by 7 dpi. For 400 µL dose group, this decrease was seen only at 14 dpi. The right lung lobes were more affected than the left lung. The foci comprised small, discrete aggregates of inflammatory cells, transitioning from primarily neutrophils to primarily macrophages after 3 days (Figure 5). In addition to the small inflammatory foci, the lungs from five animals at various timepoints in the 200 µL (*n* = 2; 1 each at 1 and 3 dpi) and 400 µL (*n* = 3; 1 each at 1, 7, 14 dpi) group also had a single focus of acute bronchopneumonia with a filling of the bronchioles and surrounding alveoli by neutrophils and macrophages. Foreign material was associated with this lesion in two animals. A Gram stain performed to assess the presence of bacteria accompanying the bronchopneumonia was negative in all cases (data not shown).

## 3. Discussion

The approaches for the delivery of respiratory instillations (aerosolization, intubation, and intranasal instillation) vary by technical requirements, efficiency of delivery to targeted location in the respiratory tract, and invasiveness. Aerosol generators are the most effective modality and can successfully target all of the aspects of the respiratory tract (RT), but require specialized machinery and consideration to ensure the agent being nebulized is introduced to the animal’s RT in its active form. Alternatively, intubation can be used to inoculate the lower RT but requires technical expertise and circumvents the nasal passages and upper RT. This method does not accurately reproduce how the infectious agents enter the RT in animals and can be invasive and produce trauma if not properly performed. IN instillation can be used to target the upper and/or lower RT, is the least technical and invasive method of the modalities discussed and allows for the active titration of the delivery substance during inoculation. Given the ease and widespread use of IN instillation for high-containment laboratories working with hamster models for respiratory pathogens, including SARS-CoV-2, we sought to generate data to guide methodology and minimize the pathological effects of the delivery approach alone that may compromise animal welfare or confound data interpretation.

Hamsters in all experimental groups, both IN-instilled and non-instilled controls, demonstrated standard growth development, the absence of respiratory signs, normal thoracic radiographs, appropriate peripheral oxygenation, and a lack of systemic inflammatory response to instillation. The pulse oxygenation of all the animals was unaffected throughout the study. A drop in oxygenation between pre- and post-instillation readings equally affected a small subset of the animals in each group, including non-instilled controls, indicating that instillation volume did not result in negative effects to peripheral tissue oxygenation. There was a notably prolonged recovery time after instillation of the animals in the 400 µL group. All animals in this group required additional monitoring, oxygenation, and stimulation during their recovery. Notably, the animals in the 400 µL group required on average 1–2 min after instillation before pulse oximetry could be obtained post-instillation, compared to 5–10 s needed for the stabilization prior to recording the first peripheral oxygenation data point in all the other groups. It is possible that the high-volume instillation led to a stress response in the animal, resulting in a vasovagal event. This would have caused decreased cardiac output and hypotension, resulting in vasodilation and decreased systemic vascular resistance [31]. Vasodilation could then have resulted in the creation of arteriovenous shunts that resulted in venous pulsations and intermittent detection by the pulse oximeter [32]. Given the side-effects immediately seen after the instillation of the 400 µL volume, lower volumes are recommended. The studies where large instillation volumes may be further examined should be vigilant of the need for increased supportive care and the monitoring of animals until full recovery.

Despite the absence of clinical signs and the maintenance of normal clinical parameters throughout the study period, we observed changes consistent with foreign material aspiration via histopathology, that increased in frequency and severity with the higher instillation volumes. No changes were seen in the lungs of non-instilled hamsters or those instilled with 50 µL. Volume-dependent histological changes were observed in a subset of animals in the 100, 200, and 400 µL groups, suggesting a volume-associated incidence of aspiration. Consistent with aspiration, the right lung lobes were more affected than the left lung lobes; the right mainstem bronchus comes off the trachea more proximally than the left and is thus the more likely location for aspirated material deposition. In a very small subset of hamsters given 100 µL, very minimal and probably insignificant changes were noted in the tissues. When the volume was increased to 200 or 400 µL, the incidence and number of foci per animal increased. Additionally, a single focus of bronchopneumonia was observed in a subset of the 200 and 400 µL animals.

These lesions could potentially confound the interpretation of pathology in models of respiratory pathogens. Later timepoint samples suggest that the lesions may resolve around 7 dpi for 200 µL and 14 dpi for 400 µL instillation groups, but true resolution cannot be confirmed as these samples were derived from different individuals at each timepoint. Furthermore, depending on the severity of bronchopneumonia, the resolution of these lesions may be prolonged or remain unresolved, potentially leading to morbidity/mortality. The origin of the foreign debris associated with inflammatory foci is not entirely clear. Given the large capacity and function of the cheek pouches in hamsters [33] and the associated normal behavior of loading feed or stuffing bedding or nesting material into their cheek pouches to carry it from one place to another, the translocation of nasal or oropharyngeal content (bedding and/or feed fragments) into the lungs by larger instillation volumes is plausible.

Altogether, these studies provide key data regarding the effects of sample volume on IN-instillation in young Syrian hamsters, with results suggesting that volumes of 100 µL or less are optimal for mitigating the IN-instillation volume-associated histological changes. Of note, our studies were in hamsters 5–6 weeks of age. In older animals, where the instillation volume to lung weight ratio would decrease correspondingly with growth, the effects of higher volume use may differ. The choice of anesthesia may also affect distribution and histological findings; inhalation (vs. injectable) anesthetics can result in a deeper distribution of larger volumes of inoculum per breath, facilitating more efficient delivery of inoculum, and possibly foreign material to the lower RT. For example, a study visualizing murine intranasal-dosing efficiency, using luminescent *Francisella tularensis*, reported that the IN-inoculated mice that were anesthetized using inhaled isoflurane had significantly higher bacterial burdens in their lungs than the mice that had been anesthetized with parenteral ketamine/xylazine [6]. Whether the use of injectable anesthesia would mitigate the histopathological changes seen here with higher volume instillation is not known. Future studies of anesthetic effects and to establish age- or weight-based IN-instillation volume recommendations in hamsters are warranted. In addition, for these studies we selected DMEM, given its widespread use in virus stock generation and for inoculum dilution in challenge models. However, composition (e.g., pH, osmotic concentration) should be considered for other diluents, as they may differentially affect the parameters examined here.

## 4. Materials and Methods

### 4.1. Intranasal Instillation in Hamsters

All animal experiments were approved by the Centers for Disease Control and Prevention (CDC) Institutional Animal Care and Use Committee and performed in an AAALAC International-approved facility. A total of 80 HsdHan:AURA Syrian hamsters (40 male, 40 female; 5 to 6-week old; average weight: 70 g, weight range: 60–83 g) were received from Envigo (no. 8903F or 8903M). The hamsters were group housed on corn cob bedding (Bed-o’Cobs^®^ ¼”, Anderson Lab Bedding, Maumee, OH, USA) with cotton nestlets and crinkle paper (Enviro-Dri^®^, Fibercore, Cleveland, OH, USA), in an isolator-caging system (Green Line, GR1800; Tecniplast, West Chester, PA, USA) with a HEPA-filtered inlet and exhaust air supply in a climate-controlled laboratory, with a 12 h day/night cycle. They were provided with Teklad global 18% protein rodent diet (Envigo, Indianapolis, IN, USA) and water ad libitum, and with seed/forage enrichment (Kaytee Forti-Diet Pro Health Hamster and Gerbil food) once every two weeks at cage changeout. Microchip transponders (BMDS IPTT-300) were placed subcutaneously in the interscapular region for individual identification and to assess body temperature. The transponder insertion site was closed using GLUture topical tissue-adhesive (Zoetis, Parsippany-Troy Hills, Morris, NJ, USA).

Groups of 16 hamsters (8 male, 8 female) were instilled intranasally (IN) under isoflurane anesthesia (induction, 2–3.5%), using a Pipet-Lite LTS Pipette L-200XLS+ (Rainin) with 50, 100, 200 or 400 µL of Dulbecco’s modified Eagle’s medium (DMEM; Gibco, REF 11965-092), divided bilaterally between the nares. A group of 16 hamsters (8 male, 8 female) served as the non-instilled controls. Animals were clinically assessed daily, and weights and temperatures were obtained. Clinical scoring included 2 points each for quiet/dull/responsive (QDR), hunched back/ruffled coat/piloerection, hypoactivity, mild respiratory signs, or mild neurological signs; 3 points each for dehydration (eye recession); 5 points each for moderate respiratory signs (dyspnea, tachypnea), moderate neurological signs (e.g., ataxia, circling, tremors, weakness or paresis), or hypothermia; and 10 points each for severe respiratory signs, severe neurological signs (e.g., paralysis), frank hemorrhage, moribund behavior, or >25% weight loss from baseline. A score of ≥10 indicated end-point criteria.

At predetermined timepoints, 4 hamsters (2 female, 2 male) from each experimental group were serially euthanized for blood and tissue analyses. A power analysis was conducted with α = 0.05, β = 0.2 to detect a difference of 1 σ by having four animals per time point and experimental condition. The subset of the animals designated at that timepoint were transported from the holding suite for final weight and temperature readings, oxygen saturation, and radiographic evaluation prior to terminal intracardiac blood collection and euthanasia by isoflurane overdose.

### 4.2. Pulse Oximetry

Oxygen saturation readings were obtained using the MouseSTAT^®^ Jr. Pulse Oximeter and Heart Rate Monitor (Kent Scientific, Torrington, CT, USA), while under isoflurane anesthesia (2–3.5%). The sensor utilized was the Rat Paw Pulse Oximeter Sensor, always placed on the hind left paw. At each timepoint, three reading replicates were obtained for each animal. The machine was allowed to equilibrate for 5 s prior to the first reading and two sequential readings were taken at 10 s intervals thereafter.

### 4.3. Complete Blood Count

Terminal intracardiac blood was collected with a 25-gauge needle and 1 mL syringe (Becton-Dickinson, Franklin Lakes, NJ, USA) under isoflurane anesthesia (2–3.5%) and immediately deposited into 1.3 mL K3E 1.6 mg EDTA/mL microtainer tubes (Sarstedt, No. 41.1395.105). The complete blood counts were performed on whole blood samples within 40 min of collection using the VetScan HM5 hematology analyzer (Abaxis, Union City, CA, USA).

### 4.4. Radiographs

Radiographs were taken with the VetPro^®^ DC (Midmark, Animal Health Dental X-ray System, Dayton, OH, USA) and evaluated with Progeny^®^ (Midmark). All animals were anesthetized in rodent boxes and transferred to an elevated cardboard platform where the digital sensor (Midmark DR Digital Dental Sensor) was housed inside for support and stability. All images were taken at 65 kVp, 7 mA, and an aperture of 0.080 s. Three-view radiographic images (ventrodorsal and laterolateral) were taken per animal prior to terminal intracardiac blood collection. All images were evaluated by two DACLAM board-certified veterinarians, one unblinded and one blinded, to assess for pulmonary changes, such as opacities, vasculature abnormalities, or infiltrates.

### 4.5. Histopathology

Necropsy was performed immediately after euthanasia. The lungs were inflated with 10% neutral-buffered formalin by insertion of a 23-gauge needle connected to a 3 mL syringe. The inflation was performed to approximate physiologic capacity. The pluck was then removed, the head was separated from the carcass, and both were fixed in formalin for 7 days. The head was subsequently decalcified for 7 days using Cal-Ex^TM^ II Fixative/Decalcifier (Fischer Chemical, Waltham, MA, USA). After decalcification, four transverse sections of the nasal cavity were taken at levels (1) caudal to the upper incisors, (2) at the incisive papilla, (3) at the second palatine crest, and (4) at the first molar teeth [34]. The nasal cavity sections, trachea, and lungs were processed by routine paraffin histology. The lungs were embedded en bloc and with the lobes splayed, so that most of the histologic sections included all lung lobes. Four micron sections were cut, stained with hematoxylin–eosin, and evaluated by two veterinary pathologists for inflammatory, degenerative, or other histopathologic changes. For the lungs, one section was examined, with the inflammatory foci counted for the entire section, including all of the lobes present.

### 4.6. Graphing and Statistical Analyses

Weight, temperature, and pulse oximetry statistics were calculated, using two-way ANOVA with Tukey’s multiple comparison test. Complete blood count statistics were calculated, using multiple *t*-tests with Holm–Siddack’s multiple comparison test. GraphPad Prism v9.3.1 software was used to generate all graphs and perform all analyses.

## Figures and Tables

**Table 1:** Summary of inflammatory foci detected in lung of Syrian hamsters. Syrian hamsters were intranasally instilled with indicated volumes of sterile Dulbecco’s modified Eagle’s medium (DMEM) and lung tissue was histologically examined at indicated timepoints post-instillation. One section was examined per animal; inflammatory foci were counted for the entire section, including all lobes present.

Volume (µL) DMEM	Day Post-Instillation	No. Inflammatory Foci (Range)
0 µL	1	0
	3	0
	7	0
	14	0
50 µL	1	0
	3	0
	7	0
	14	0
100 µL	1	0–1 *
	3	0–2 *
	7	0–3 *
	14	0–2 *
200 µL	1	1–2
	3	2–5
	7	0–2
	14	0–1
400 µL	1	6–12
	3	1–3
	7	4–5
	14	0–1

* Foci only detected in one animal from group at designated timepoint.
